# Combined Lutein and Exercise Intervention Alleviates Brain Alteration Induced by a High‐Fat Diet in Obese Rats

**DOI:** 10.1002/fsn3.71913

**Published:** 2026-05-22

**Authors:** Saraa A. Almutairi, Hanna M. Alhoraibi

**Affiliations:** ^1^ Department of Biochemistry, Faculty of Science King Abdulaziz University Jeddah Saudi Arabia; ^2^ Pre‐Clinical Research Unit, King Fahd Medical Research Center King Abdulaziz University Jeddah Saudi Arabia

**Keywords:** brain, high‐fat diet, lutein, obesity, physical exercise

## Abstract

Obesity is a major global health issue, causing many diseases in organs like the liver, heart, and brain. Diverse strategies, including dietary interventions and regular exercise, are potential strategies to combat obesity and its obesogenic consequences on the brain. Natural products like carotenoids such as lutein (Lu) have several positive impacts on human health and can help in reducing obesity. This study aimed to evaluate the combined effects of lutein supplementation and physical exercise (PE) on metabolic and hippocampal alterations associated with obesity. Wistar rats were fed a standard diet as a negative control group, and the other group was fed a high‐fat diet (HFD) for 12 weeks. Then, obese rats were randomly divided into four groups based on the following treatments: (i) a HFD only, (ii) a high‐fat diet supplemented with lutein (HFD + Lu), (iii) a high‐fat diet plus physical exercise (HFD + PE), (iv) a high‐fat diet plus a combination of lutein and physical exercise (HFD + Lu + PE) for another 5 weeks. By the end of Week 17, all groups went through a behavioral test (Y‐maze), and blood samples, visceral and brain tissues were collected for biochemical and histological examinations, respectively. Our results showed that combining Lu and PE significantly reduced blood glucose, lipid profile parameters, leptin, and malondialdehyde (MDA) levels compared with the HFD group (all *p* < 0.0001). Furthermore, the HFD + Lu + PE group exhibited significantly higher levels of high‐density lipoprotein (HDL‐C), catalase (CAT), and superoxide dismutase (SOD), along with significantly lower levels of interleukin‐6 (IL‐6) compared with the HFD group (all *p* < 0.0001). Treatments with Lu, PE, and Lu + PE significantly enhanced short‐term spatial memory performance in the Y‐maze (*p* < 0.001) and attenuated hippocampal histopathological alterations compared with the HFD group. This study demonstrated that combining lutein and physical exercise is an alternate approach for improving brain health and preventing obesity and its complications in rats fed with HFD. Additionally, the combination of Lu and PE had more beneficial effects on the rat's brain than either Lu or PE alone.

## Graphical abstract

High‐fat diet‐induced obesity caused metabolic dysfunction, oxidative stress, inflammation, and hippocampal impairment in Wistar rats. The combination of lutein supplementation and physical exercise improved metabolic markers, reduced inflammation and oxidative stress, enhanced antioxidant defense, and attenuated memory impairment and hippocampal damage compared with the high‐fat diet group. The combined treatment showed greater neuroprotective and anti‐obesity effects than lutein supplementation or physical exercise alone.

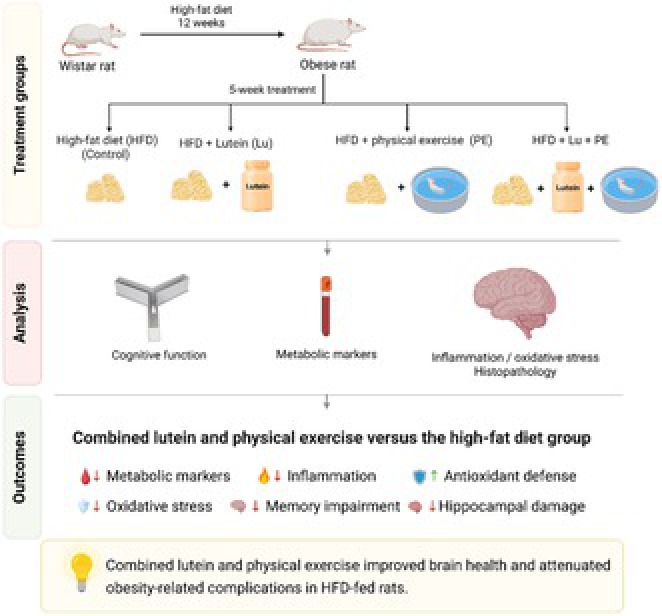

**Abbreviations**
ANOVA: analysis of variance
Bc: blood capillaries
BDNF: brain‐derived neurotrophic factor
BW: body weight
CA: Cornu Ammonis
CAT: catalase
CNS: central nervous system
COX‐2: cyclooxygenase‐2
DG: dentate gyrus
ELISA: enzyme‐linked immunosorbent assay
GC: granule cells
GCL: granular layer
HDL‐C: high‐density lipoprotein cholesterol
HFD: high‐fat diet
IL‐1β: interleukin‐1 beta
IL‐6: interleukin‐6
iNOS: inducible nitric oxide synthase
KFMRC: King Fahd Medical Research Centre
kg/m^2^
: kilogram per square meter
LDL‐C: low‐density lipoprotein cholesterol
Lu: lutein
MDA: malondialdehyde
ML: molecular layer
NADPH: nicotinamide adenine dinucleotide phosphate
NF‐κB: nuclear factor kappa B
NOX: NADPH oxidase
Nrf2: nuclear factor erythroid 2‐related factor 2
PC: pyramidal cells
PCL: pyramidal cell layer
PE: physical exercise
PML: polymorphic layer
PPARα: peroxisome proliferator‐activated receptor alpha
ROS: reactive oxygen species
RPM: revolutions per minute
SA: spontaneous alternation
SD: standard deviation
SOD: superoxide dismutase
TC: total cholesterol
TG: triglyceride
TNF‐α: tumor necrosis factor alpha
VAT: visceral adipose tissue
VLDL‐C: very low‐density lipoprotein cholesterol

## 1 Introduction

Obesity is a major global health concern and was declared an epidemic by the World Health Organization in 1997 (Ayalon et al. 2021). It arises from complex interactions between genetic, environmental, and behavioral factors, including physical inactivity and unhealthy dietary patterns (Cai et al. 2023). Beyond its well‐established metabolic consequences, obesity is increasingly recognized for its detrimental effects on the brain, contributing to structural and functional alterations, heightened risk of dementia, and cognitive decline (Livingston et al. 2020; Sui and Pasco 2020). Experimental studies further demonstrate that excess adiposity impairs learning and memory (Farr et al. 2008). Mechanistically, obesity‐associated hyperglycemia, dyslipidemia, vascular dysfunction, and high‐fat diet (HFD)–induced neuroinflammation and oxidative stress collectively disrupt central nervous system (CNS) integrity and metabolic homeostasis (Mullins et al. 2020).

Obesity poses significant health challenges and has negative impacts on overall quality of life. Subsequently, developing preventive treatments is a crucial step toward maintaining comprehensive health. A broad range of strategies is recommended to reduce the prevalence of obesity. Due to the unfavorable side effects of anti‐obesity medications or surgical treatments, the combined impact of regular exercise and food modification continues to be the most secure and economical way to manage obesity (Ammar et al. 2017). Furthermore, natural bioactive compounds such as carotenoids have gained increasing attention for their potential role in mitigating obesity and its metabolic complications (Gopal et al. 2023).

Among these natural products, carotenoids have attracted considerable research interest due to their diverse biological activities. Carotenoids are fat‐soluble pigments classified into two main types based on their chemical structure: carotenes (hydrocarbons like β‐carotene and lycopene) and xanthophylls (polar compounds with oxygen atoms in their molecules like lutein and its stereoisomer zeaxanthin) (Fiedorowicz and Dobrzyńska 2023; Prathyusha et al. 2025). Lutein, a xanthophyll carotenoid found abundantly in egg yolk and leafy green vegetables (Ahn and Kim 2021), is particularly noteworthy. It is the second‐most prevalent carotenoid in human serum and cannot be synthesized by the human body, making dietary intake essential (Turk et al. 2020). Lutein exhibits multiple biological activities relevant to obesity and its complications. It possesses potent antioxidant properties, scavenging reactive oxygen species such as singlet oxygen and lipid peroxyl radicals (Ahn and Kim 2021). In addition, lutein shows anti‐inflammatory, anticancer, and antimicrobial effects in different experimental models (Ahn and Kim 2021; Fiedorowicz and Dobrzyńska 2023). Importantly, lutein has demonstrated anti‐adipogenic effects by suppressing lipogenesis, reducing intracellular lipid accumulation and adipose tissue weight, and improving dyslipidemia (Liu et al. 2017; Seo et al. 2016).

Beyond its metabolic effects, lutein contributes to cardiovascular, cognitive, and ocular health. Evidence suggests that lutein supports cognitive function and helps maintain hippocampal integrity, particularly under metabolic stress (Liu et al. 2025; Cannavale et al. 2019). Lutein preferentially accumulates in neural tissues and has been associated with cognitive performance (Erdman et al. 2015; Jain et al. 2026). It has also been reported to cross the blood–brain barrier and localize within neuronal membranes, where it may modulate oxidative and inflammatory pathways linked to neural efficiency (Erdman et al. 2015; Cannavale et al. 2019). Therefore, lutein was chosen among carotenoids in this study due to its antioxidant, anti‐inflammatory, metabolic, and neuroprotective properties, which are directly relevant to obesity and its associated complications.

Physical exercise plays a crucial role as both a treatment and preventive measure for various illnesses, including osteoarthritis, obesity, Alzheimer's disease, and hypertension (Wang et al. 2020). Exercise enhances the insulin sensitivity of adipose tissue, decreases total and visceral adipose depots, raises lean mass index, and increases energy consumption during rest. The exercise also enhances endothelial function, which lowers triglyceride (TG) and low‐density lipoprotein cholesterol (LDL‐C) levels, raises high‐density lipoprotein cholesterol (HDL‐C), as well as decreases free fatty acids, metabolic and cardiovascular problems. Exercise serves as a catalyst for efficiently utilizing stored energy sources and promoting weight loss over time (Bülbül 2020). Research shows that regular exercise, including aerobic and resistance training, effectively lowers inflammatory markers in obese individuals. Moderate exercise regulates immune function, reduces chronic inflammation, and enhances metabolic health related to obesity (Guo et al. 2024). Physical activity has a significant positive effect on brain function and mental well‐being. Both animal and human studies have demonstrated that exercise induces positive effects on cognition through various mechanisms. These include alterations in brain volume and connectivity, enhanced cerebral perfusion, synaptic plasticity, neurogenesis (the birth of new neurons), and the regulation of neurotrophic factors: (1) neurotrophins; (2) glial cell‐line‐derived neurotrophic; (3) neuropoietic cytokines. These neurotrophic factors are induced by muscle activity and they drive changes that support the development of a more flexible and adaptable brain. Ultimately, exercise helps in maintaining the structure and function of the brain (Chen et al. 2023; Cabral et al. 2019; Blazer et al. 2015).

Accordingly, lutein and physical exercise were selected due to their reported anti‐inflammatory, antioxidant, and metabolic regulatory properties, making them biologically plausible interventions for targeting obesity‐related brain dysfunction. To comprehensively characterize obesity‐induced dysfunction, metabolic (glucose, lipid profile, and leptin), inflammatory (IL‐6), and oxidative stress (MDA, SOD, and CAT) markers were selected, as these represent key interconnected pathways linking adiposity to cognitive impairment and neuroinflammation (Naomi et al. 2023).

At the present time, few if any detailed investigations into the impact of lutein and exercise on the brain of the obese have been published. Consequently, this study aimed to evaluate the combined effects of lutein supplementation and physical exercise on obesity‐induced metabolic and hippocampal alterations. We hypothesized that the combined intervention is expected to provide complementary protection by attenuating inflammation and oxidative stress.

## 2 Materials and Methods

### 2.1 Animals and Experimental Design

Fifty male Wistar rats aged 7 weeks, weighing 200 ± 10 g, were obtained from the animal house unit of King Fahd Medical Research Centre (KFMRC), Jeddah, Saudi Arabia. The rats were acclimatized for 7 days under controlled environmental conditions (23°C ± 2°C, 60% ± 5% humidity, 12:12 h light–dark cycle). The animals were housed in standard polycarbonate cages with stainless steel wire lids at a density of five rats per cage, with wood shaving bedding changed regularly. Rats had free access to their respective diets and water throughout the study. Food intake was calculated per cage by weighing the amount of diet provided and the remaining food. The animal protocol for this study was approved by the Ethics Committee of King Abdulaziz University (Approval No. ACUC‐21‐09‐37) and followed the rules and regulations of the Animal Care and Use Committee (ACUC) at the KFMRC.

**FIGURE 1:**
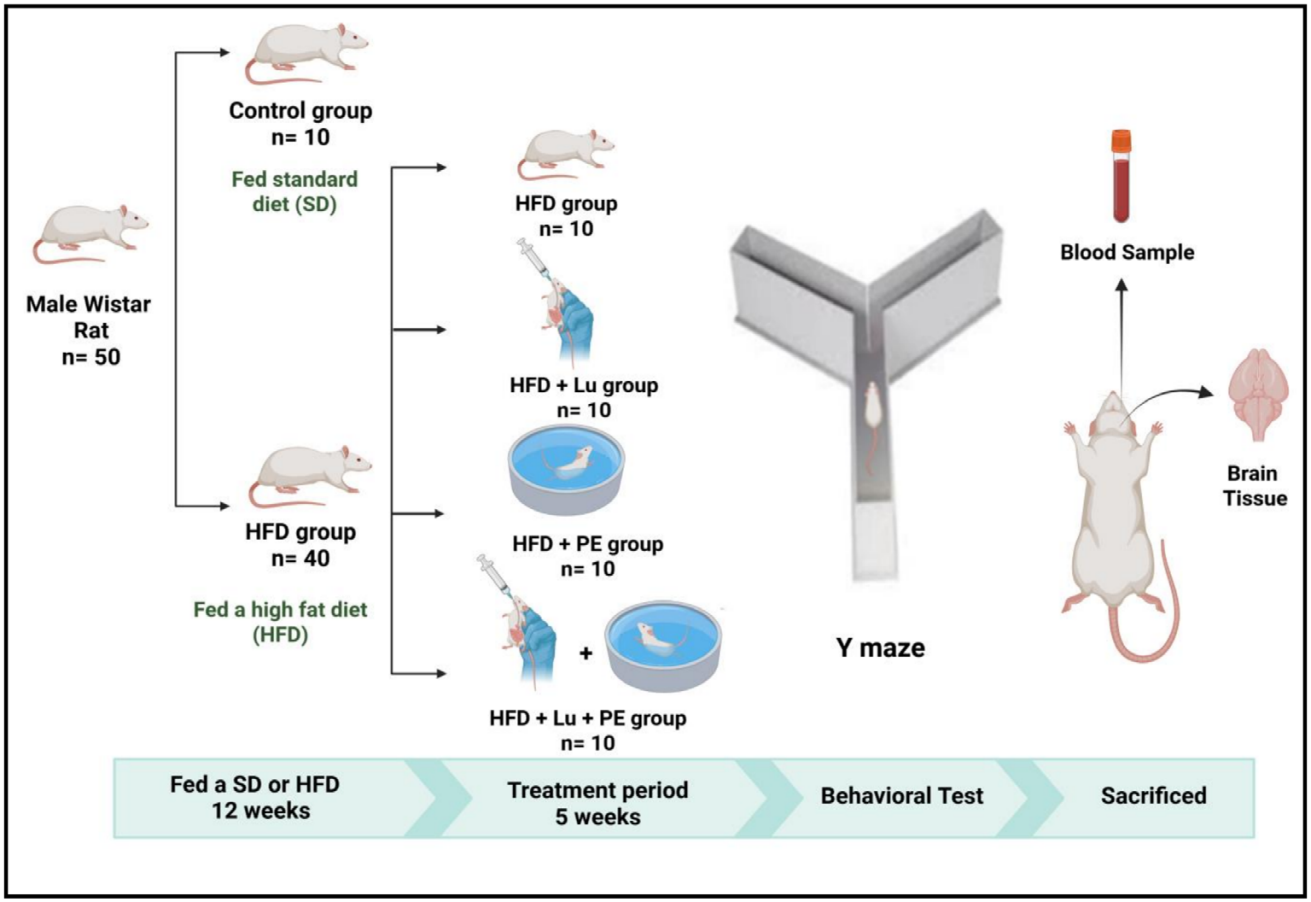
Study design. The study design showing the distribution of rats into study groups: Control group fed standard diet (SD), high‐fat diet group (HFD), high‐fat diet + lutein group (HFD + Lu), high‐fat diet + physical exercise group (HFD + PE), and high‐fat diet + lutein + physical exercise group (HFD + Lu + PE). This figure was made using the BioRender software.

The study design was depicted in Figure 1. After a 1‐week acclimatization period, 50 rats were divided into two initial groups: a negative control group (*n* = 10) fed a standard diet and an obesity group (*n* = 40) fed a HFD for 12 weeks to induce obesity. After obesity induction, the 40 HFD‐fed rats were randomly assigned into four subgroups (*n* = 10 each): (i) HFD (positive control), (ii) HFD + lutein (Lu), (iii) HFD + physical exercise (PE), and (iv) HFD + Lu + PE. All obese groups continued receiving the HFD for an additional 5 weeks during the treatment period, resulting in a total experimental duration of 17 weeks. Lutein was administered as previously described by Narayanamurthy et al. (2018). While sunflower oil was used as a vehicle for lutein, all groups were given the same amount of sunflower oil (2 mL/kg/day) during the experiment (Pierine et al. 2014; Sindhu et al. 2010). By the end of the 17 weeks, all groups underwent a behavioral test (Y‐maze) (Alghamdi 2022).

### 2.2 Diet and Exercise Intervention

Standard diet for this study was obtained from KFMRC. The diet consists of crude fat (4%), crude protein (20%), natural fiber (3.50%), choline chloride (0.25%), mixed vitamins (1%), and mixed minerals (3.50%), that were mixed with corn starch to complete (100%) and with energy equals to 2850 kcal/kg (percent by weight). The diet was acquired from Grain Silos & Flour Mills Organization in KSA. The HFD with a total caloric value of (529 kcal/100 g) was made by mixing (DL‐methionine 0.2%, casein 7.45%, commercial butter 29.79%, and vitamins and minerals 2.97%) with the standard diet 59.6% (Al‐thepyani et al. 2022; Gheibi et al. 2017). Lutein (50 mg/kg/day) derived from marigold flowers from (Xi'an Tongze Biotech Co, China) was mixed with sunflower oil (2 mL/kg/day) and commercial cow butter and stored at 4°C in the dark until use (Sindhu et al. 2010).

Swimming was employed as a model exercise intervention in this study. The HFD + PE and HFD + Lu + PE swimming groups of rats swam in a 78 × 56 × 48 cm plastic swimming pool. The water was deep enough to forbid relaxing and to hinder bobbing. The water was heated to 32°C ± 2°C to prevent hypothermia. On the first day, the rats swam for 10 min. Every day, the exercise period was extended by 10 min. The rats were given permission to swim for 60 min once daily for the next 5 weeks (Al‐thepyani et al. 2022; Maigoda et al. 2016; Riyahi et al. 2016).

### 2.3 Behavioral Test

The Y‐maze test is used to evaluate rodents' short‐term spatial working memory (Bahaidrah et al. 2022). Additionally, the basic Y‐maze test may be used to examine spontaneous alternation behavior in this form of memory (Alghamdi 2022). It has three identical arms, each measuring 18 cm in width, 40 cm in height, and evenly spaced at 120°. To put it briefly, rats were positioned in one Y‐maze arm that faced the wall, and after 8 min, entry into the other arms was noted (Muhammad et al. 2019). When the rat went to each of the three arms one after the other without repeating, it was called an alternation. The percentage of spontaneous alternation was calculated as follows: % alternation = number of alternations/(total arm visits−2); memory impairment was linked to a lower proportion of spontaneous alternation activity (Bahaidrah et al. 2022). In the Y‐maze behavioral test, the sample size was reduced (*n* = 6 per group) because some animals did not complete the test protocol and were excluded from the final analysis.

### 2.4 Assessment of Body Weight, Food Consumption, and Calorie Intake

Body weight (BW) of all experimental rats was recorded weekly and food consumption was monitored daily throughout the experimental period. The food consumption was calculated by deducting the food leftovers from the amount of food offered and dividing it by the number of cages. Calorie intake was estimated based on 9 kcal/g for lipids and 4 kcal/g for protein and carbohydrates. Calorie intake was calculated by multiplying the weight of food eaten by the caloric content per gram (Gong et al. 2016).

### 2.5 Relative Weight of Visceral Adipose Tissue

The weight of visceral adipose tissue of all rats was measured at the end of the experiment. The ratios of the visceral adipose tissue weight to body weight were represented as relative weight per 100 g of body weight (Yang et al. 2018).

Relative weights of visceral adipose or brain tissues were calculated using the following equation:

$$\text{Relative organ weight}\;\left(\%\right)=\left(\text{organ weight}\right)/\left(\text{final body weight}\right)\times 100$$

### 2.6 Surgical Procedures

Prior to sacrificing, 12‐h fasting rats were anesthetized with isoflurane and blood specimens (retro‐orbital sinus) were collected. Brain and visceral adipose tissues were immediately removed and weighed at the end of the experiment. A part of each brain was saved in formalin fixative for histology, while the remaining tissue was stored at −80°C until analysis.

### 2.7 Serum Biochemical Analysis

Serum was obtained by centrifugation (3000 rpm for 15 min) and kept at −80°C until they were required for analysis. The levels of glucose, serum TG, total cholesterol (TC), LDL‐C, and HDL‐C were quantified in King Fahad Armed Forces Hospital, Jeddah, Saudi Arabia, using standard diagnostic kits with an automated modular analyzer COBAS 8000 series. The level of very low‐density lipoprotein (VLDL‐C) was calculated based on Friedewald's equation (Friedewald et al. 1972): VLDL‐C = TG/5. The serum leptin level was determined via an enzyme‐linked immunosorbent assay (ELISA) kit following the manufacturer's instructions (Solarbio Science & Technology Co., Beijing, China).

### 2.8 Brain Sample Analysis

Spectrophotometer kits were used to test the levels of antioxidant markers superoxide dismutase (Cat. No: BC0175) and catalase (Cat. No: BC0205) and oxidative stress markers malondialdehyde (Cat. No: BC0025) in the homogenized brain tissue. The ELISA kits were used to measure the levels of inflammatory markers IL‐6 (Cat. No: SEKR‐0005) in the brain tissue according to the manufacturer's instructions. All kits were purchased from (Solarbio Science & Technology Co., Beijing, China).

### 2.9 Histological Analysis of Hippocampal Tissues

At the end of the experiment, to investigate the histological change in the brain after treatment, rats were anesthetized and sacrificed. Brain tissues were collected, rinsed immediately with normal saline, and placed in 10% buffered formalin fixative for 24 h. Paraffin‐embedded blocks were sagittally sectioned at 2–5 μm thickness. Following that, slices were photographed at ×400 magnification and stained with hematoxylin and eosin for histological analysis under a light microscope.

### 2.10 Statistical Analysis

Data are presented as mean ± SD. Individual data points are overlaid on the bar graphs to illustrate within‐group variability. Statistical analyses were performed using GraphPad Prism (version 9.4.1). Endpoint measurements were analyzed using one‐way Analysis of variance (ANOVA) followed by Tukey's multiple comparisons test. Food and energy intake were analyzed as repeated measurements using a mixed‐effects model (REML), with time as a repeated measure and group as a fixed factor; cage was considered the experimental unit. A value of *p* < 0.05 was considered statistically significant.

## 3 Results

### 3.1 Effect of Lutein and Physical Exercise in Preventing HFD‐Induced Adiposity

**FIGURE 2:**
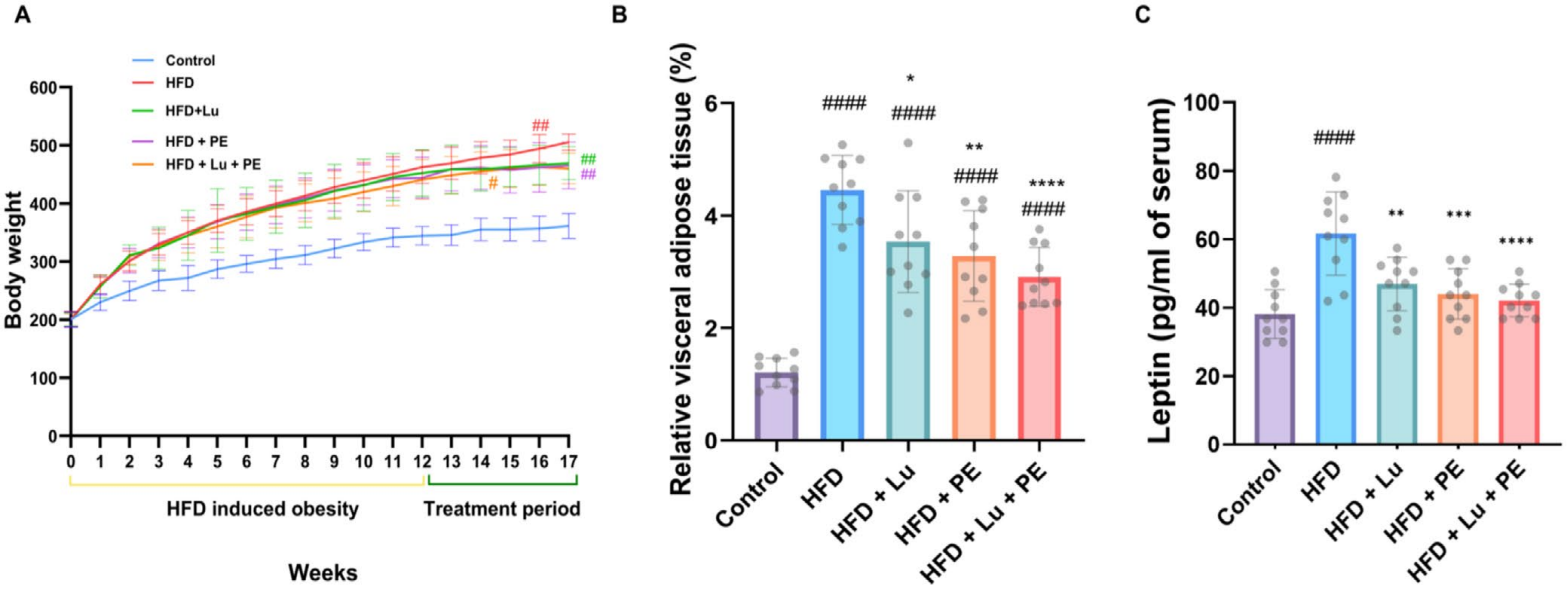
Lutein and physical exercise ameliorate the HFD‐induced adiposity. (A) Body weight of HFD‐fed rats over 17 weeks. (B) Weight of relative visceral adipose tissue; (C) Leptin level. Data are presented as mean ± SD, *n* = 10. Significant at ^#^ *p* < 0.05, ^##^ *p* < 0.01, ^####^ *p* < 0.0001 vs. control, *p < 0.05, **p < 0.01, ***p < 0.001, ****p < 0.0001 versus HFD.

All rats started the treatment with similar weights. The increase in body weight over 17 weeks was statistically higher in groups fed HFD in comparison to control (Figure 2A). The final body weight in the HFD + Lu + PE group is significantly decreased compared to the HFD group. Likewise, both groups treated with Lu or PE are significantly decreased compared with the HFD group. Moreover, weight gain was significantly increased in the HFD group compared to the control; however, the effect of Lu and/or PE on weight gain was not significant compared to the HFD group as seen in Table 1. Food consumption was slightly higher in the control group compared to the HFD group; however, the difference was not statistically significant. Similarly, caloric intake was slightly higher in the HFD group compared to the control group, but this difference did not reach statistical significance. No significant differences in food or energy intake were observed among the HFD‐treated groups.

**TABLE 1:** The effect of Lu and PE on body weight, food consumption, and calorie intake in HFD‐fed rats.

Parameter	Control	HFD	HFD + Lu	HFD + PE	HFD + Lu + PE
Initial body weight (g)	200.50 ± 12.40	200.90 ± 12.73	200.60 ± 11.43	200.80 ± 13.84	200.10 ± 10.40
Final body weight (g)	361.40 ± 21.57	505.50 ± 13.83***	469.40 ± 28.21*	465.60 ± 40.05*	460.40 ± 26.07**
Weight gain (%)	80.68 ± 13.27	152.50 ± 15.04***	134.90 ± 20.72	132.50 ± 21.23	130.60 ± 16.46
Food consumption (g/day)	27.33 ± 0.26	27.98 ± 0.49	27.10 ± 1.50	27.27 ± 0.003	27.02 ± 0.46
Calorie intake (kcal/day)	105.78 ± 1.00	148.01 ± 2.61	143.35 ± 7.94	144.24 ± 0.02	142.79 ± 2.42

*Note:* Data are presented as mean ± SD. Rats per group (*n* = 10) for body weight parameters; food consumption and caloric intake were calculated per cage (*n* = 2 cages per group). Significant at *p < 0.05, **p < 0.01 versus HFD, ***p < 0.0001 versus control.

The visceral adipose tissue (VAT) weight and serum leptin levels are two parameters that are linked to obesity and its complications. Data in Figure 2B demonstrated that the HFD diet induced higher visceral adipose tissue weight compared to control rats. However, the weight of visceral adipose tissue in the groups that consumed HFD and were treated with Lu, PE, and Lu + PE was remarkably lowered compared to the HFD group. Furthermore, as shown in Figure 2C, the leptin level was significantly greater in the HFD group compared with the control, whereas the groups (HFD + Lu, HFD + PE, and HFD + Lu + PE) showed lower leptin levels compared to the HFD group. Combined Lu and PE have a greater effect on VAT weight and leptin levels than the effects of either Lu or PE alone.

### 3.2 Lutein and Physical Exercise Ameliorate Lipid Profile and Glucose in HFD‐Fed Rat

**FIGURE 3:**
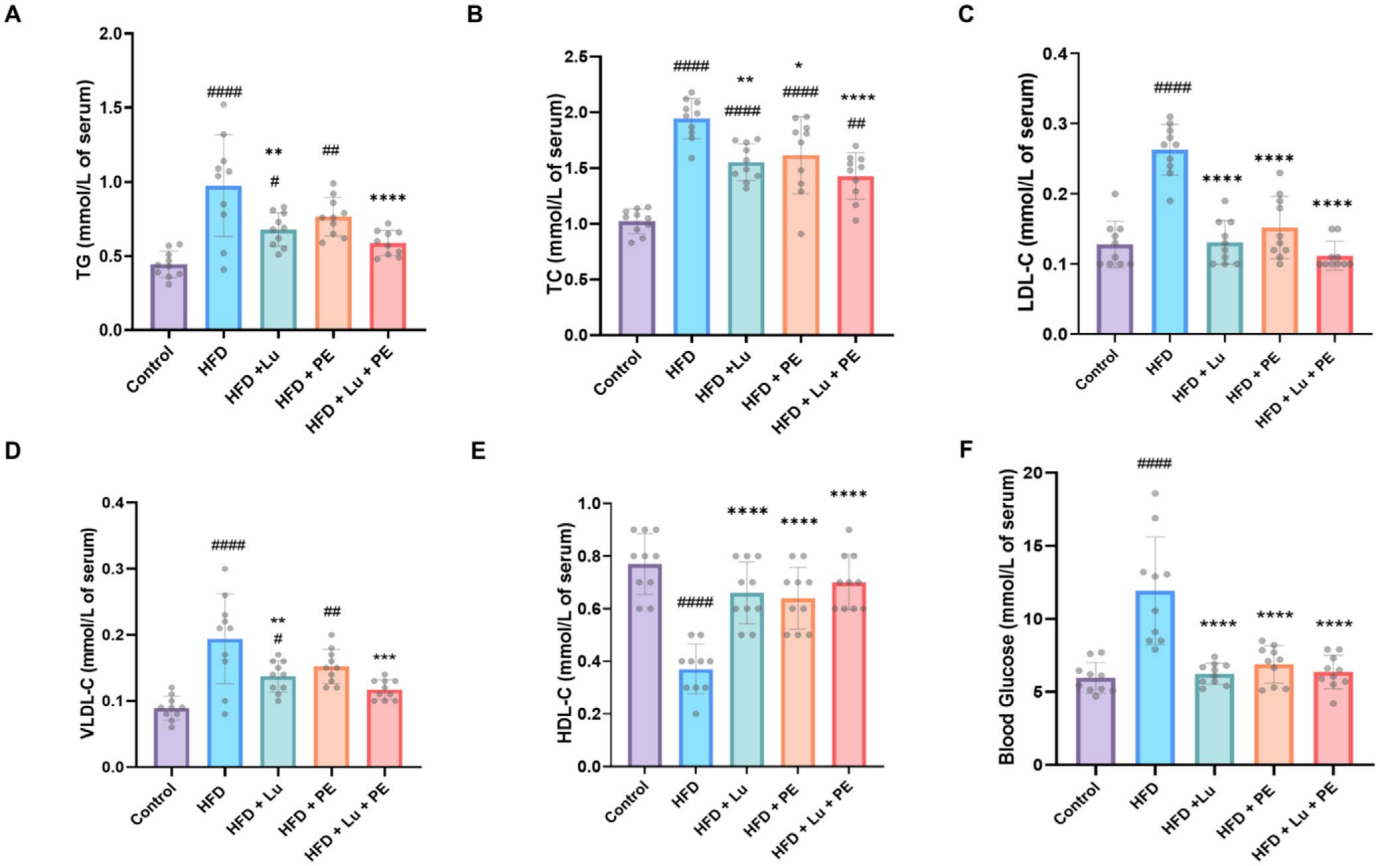
Lutein supplementation and physical exercise improve lipid profile and reduce blood glucose levels in obese rats. (A) Triglyceride (TG), (B) total cholesterol (TC), (C) low‐density lipoprotein (LDL‐C), (D) very low‐density lipoprotein (VLDL‐C), (E) high‐density lipoprotein (HDL‐C), (F) blood glucose level. Data are presented as mean ± SD, *n* = 10. Significant at ^#^ *p* < 0.05, ^##^ *p* < 0.01,^####^ *p* < 0.0001 versus control, *p < 0.05, **p < 0.01, ***p < 0.001, ****p < 0.0001 versus HFD.

As HFD could affect lipid metabolism and cause hyperglycemia, the serum lipid indexes (TG, TC, LDL‐C, VLDL‐C, and HDL‐C) and blood glucose levels were measured (Al‐thepyani et al. 2022). Figure 3 represents the significantly higher level of TG, TC, LDL‐C, VLDL‐C, and glucose in the HFD group compared to the control. There was a significant lowering in TG, TC, LDL‐C, and VLDL‐C levels in the groups consumed HFD and treated with Lu and/or PE in comparison with the HFD (Figure 3A–D). Also, the level of HDL‐C was significantly lower in the HFD group compared with the control as shown in Figure 3E. After treatment with lutein and/or doing exercise, the HDL‐C was significantly higher compared with the HFD group. Moreover, the blood glucose levels were significantly lowered in the Lu and/or PE groups compared with the HFD group as shown in Figure 3F. The combination of Lu and PE significantly enhanced the levels of lipid profile and glucose than either Lu or PE alone (Table S1).

### 3.3 Lutein and Physical Exercise Attenuate Oxidative Stress and Inflammatory Response Induced by HFD in Obese Rat's Brain

**FIGURE 4:**
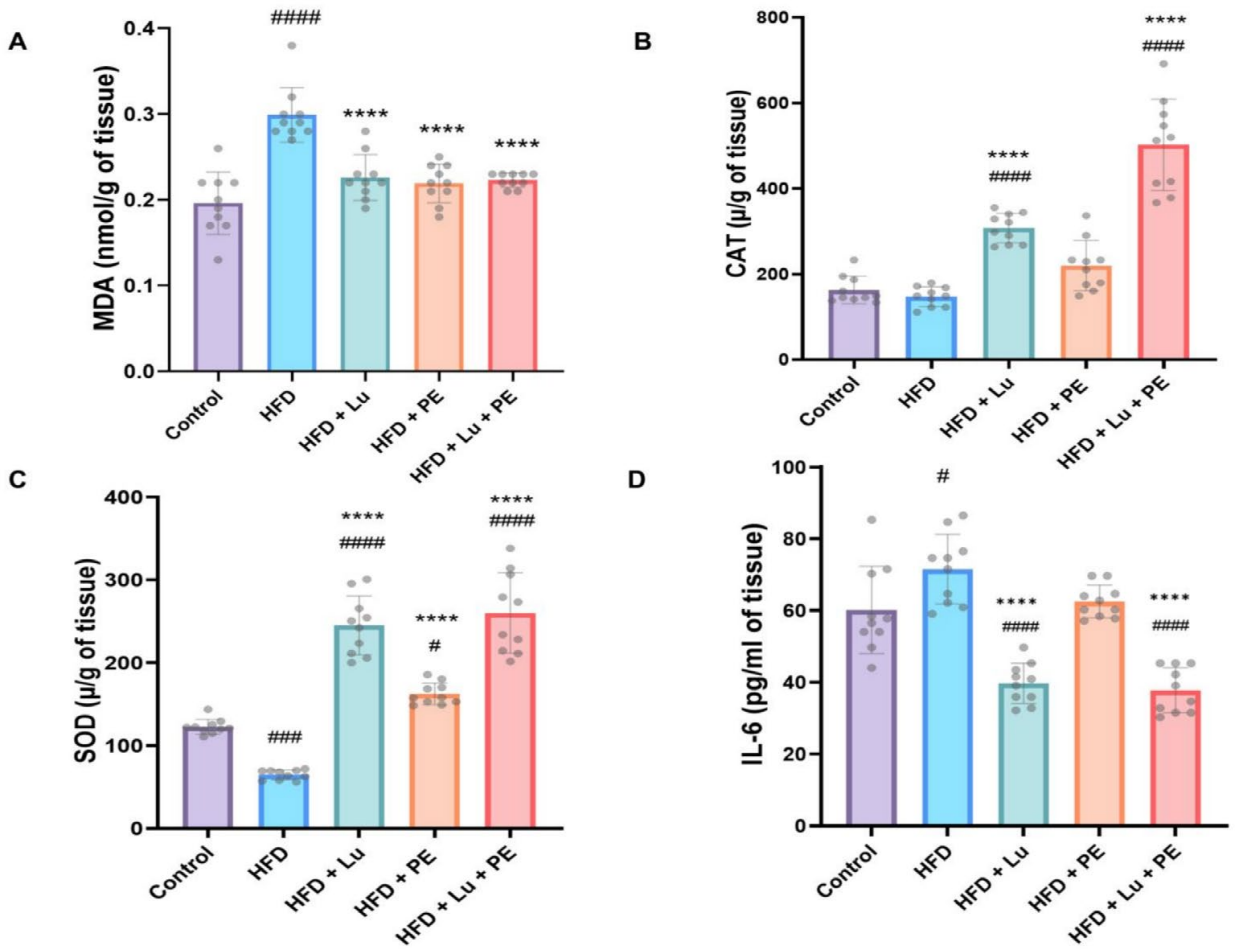
Lutein supplementation and physical exercise suppress oxidative stress and inflammatory response in the brains of obese rats. (A) Oxidative stress was assessed by malondialdehyde (MDA), (B) Antioxidant enzyme activities by catalase (CAT), and (C) Superoxide dismutase (SOD); (D) Inflammation was evaluated by Interleukin‐6 (IL‐6). Data are presented as mean ± SD, *n* = 10. Significant at ^#^ *p* < 0.05, ^###^ *p* < 0.001, ^####^ *p* < 0.0001 versus control, ****p < 0.0001 versus HFD.

A HFD can lead to obesity, which causes oxidative stress by increasing lipid peroxidation and reducing antioxidant activity (El Ayed et al. 2017). Figure 4A shows that the malondialdehyde (MDA) level was significantly higher in the HFD group compared with the control. By comparing all treatment groups with the HFD group, the MDA level was significantly lower. Additionally, the catalase (CAT) activity level revealed that the HFD group showed no significant change compared to the control group. Moreover, the HFD + Lu and HFD + Lu + PE groups showed a significant higher CAT level compared with the HFD group (Figure 4B). Also, the SOD level in the HFD group was significantly decreased compared with the control. By comparing the groups (HFD + Lu, HFD + PE, and HFD + Lu + PE) with the HFD group, the levels of SOD were significantly higher (Figure 4C). The combination of Lu and PE significantly lowered oxidative stress and enhanced antioxidant activity more than either Lu or PE alone (Table S1).

Chronic obesity is also associated with inflammation, which can negatively impact cognitive function, particularly memory (Labban et al. 2020). Interleukin‐6 levels in Figure 4D showed a significantly higher level in the HFD group compared with the control. It was noted that both groups, HFD + Lu and HFD + Lu + PE exhibited significantly lower values compared with the HFD group (Table S1).

### 3.4 Effect of Lutein and Physical Exercise on Behavioral Test and Hippocampus Morphology

**FIGURE 5:**
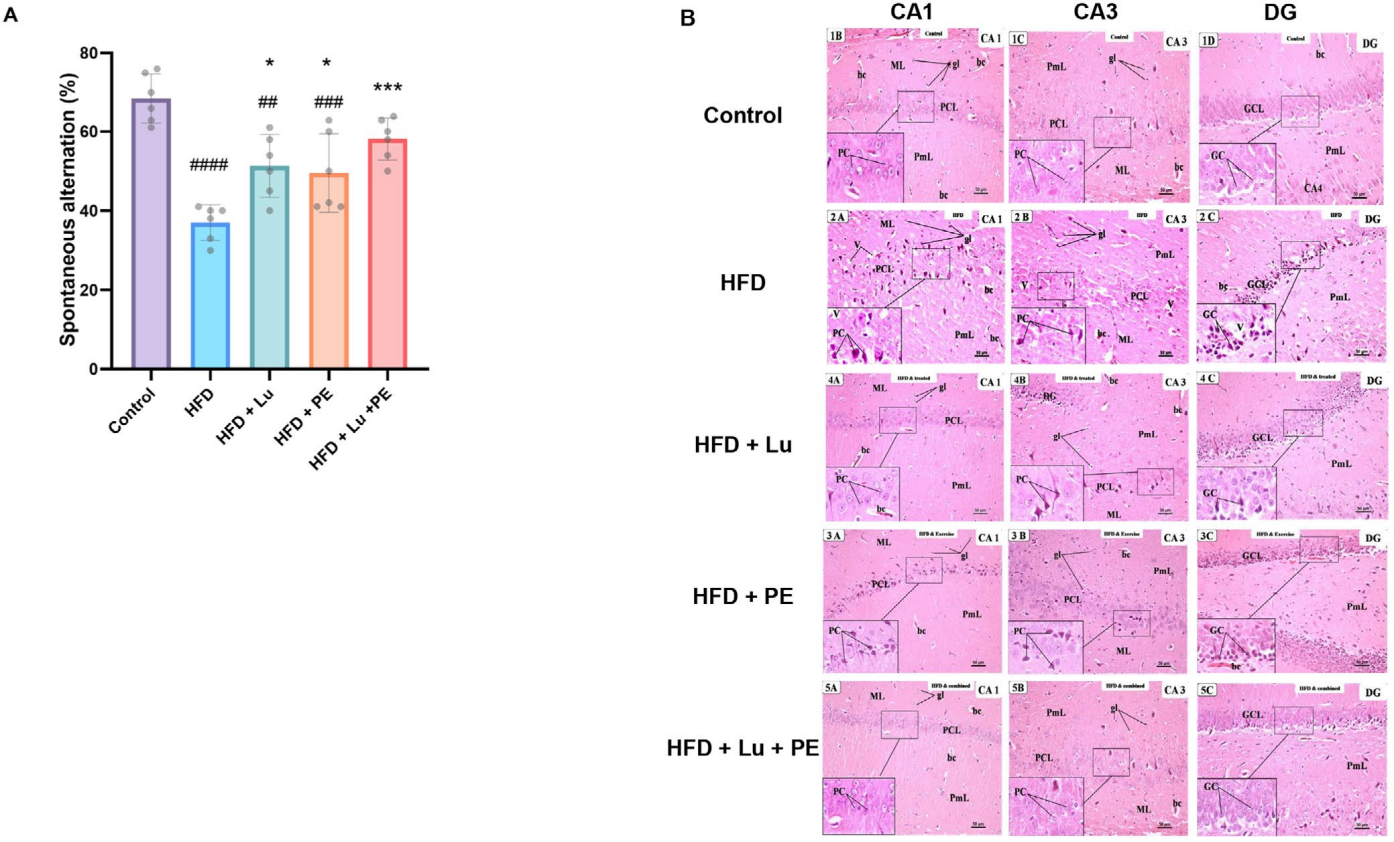
Effects of Lu and PE on behavioral test and hippocampus morphology. (A) Spontaneous alternation (%) in the Y maze. (B) Hippocampus morphology. Data are presented as mean ± SD, *n* = 6. Significant at ^##^ *p* < 0.01, ^###^ *p* < 0.001, ^####^ *p* < 0.0001 versus control, *p < 0.05, ***p < 0.01 versus HFD. Photomicrographs of H & E‐stained brain parasagittal sections. [×200, Inset ×400].

Obesity induces alterations in brain morphology and function, leading to impairments in cognition, learning, and memory (Livingston et al. 2020; Sui and Pasco 2020). The Y‐maze test was performed to measure spatial memory. The Y‐maze result in Figure 5A shows significantly lower spontaneous alternation (%) in the HFD group compared with the control. After HFD was treated with lutein (Lu) and/or exercise (PE), the three treated groups showed a significantly higher spontaneous alternation compared with the HFD group. The combination of Lu and PE resulted in significantly higher spontaneous alternation than either Lu or PE alone.

The examination of the brain sections from the control group showed a normal shape and structure of the hippocampus, which included Cornu Ammonis (CA) and dentate gyrus (DG). Cornu Ammonis had a C‐shaped body that comprised CA1, CA2, CA3, and CA4 regions. Both CA1 and CA3 were composed of three well‐defined layers: an outer molecular layer, a middle pyramidal cell layer, and an inner polymorphic layer (Figure 5B). The pyramidal cell layer (PCL) was the most prominent layer and was formed of several layers of densely packed small pyramidal cells in CA1 and less densely packed large pyramidal cells in CA3. The pyramidal cells (PC) displayed pale basophilic cytoplasm and large rounded vesicular nuclei. The molecular (ML) and polymorphic (PmL) layers consisted of pinkish neuropil that contained a few scattered neuroglial cells and blood capillaries (BC) (Figure 5B). The DG had a V‐shaped structure enclosing the CA4 region by upper and lower limbs. Each limb was composed of three layers: an outer molecular layer, an intermediate granular layer, and an inner polymorphic layer. The granular layer consisted of closely packed small granular cells with rounded nuclei (Figure 5B).

In the HFD group, variable histopathological changes were observed in different regions of the hippocampus. The main alterations were seen in the pyramidal cell layer of CA1 and CA3, which showed a disorganized arrangement compared with the control group. Many pyramidal cells showed degenerative changes, having lost their shape and appearing shrunken with dark cytoplasm and condensed nuclei. Both the ML and PmL showed increased neuroglial cells, dilated blood capillaries, and vacuoles in the neuropil. In the DG, the main changes occurred in the granular cell layer, which appeared disorganized, with many granular cells showing degeneration and shrinkage, along with vacuolated cytoplasm and condensed nuclei. The ML and PmL of the DG also showed increased neuroglial cells with pericellular haloes and dilated blood capillaries (Figure 5B).

Compared with the HFD group, the treated groups showed different degrees of histopathological improvement. The HFD + Lu and the HFD + PE groups showed histopathological improvement, with relatively normal thickness of the pyramidal layer in both CA1 and CA3, although some pyramidal cells still appeared shrunken with dark condensed nuclei. The molecular and polymorphic layers also showed increased glial cells and some dilated blood capillaries, while the DG showed moderate improvement, although some granular cells still appeared shrunken with condensed nuclei. The HFD + Lu + PE group showed marked improvement, with relatively normal thickness and arrangement of the pyramidal layer in CA1 and CA3, where most pyramidal cells appeared preserved in shape and showed vesicular nuclei. The molecular and polymorphic layers showed fewer glial cells and some dilated blood capillaries. The DG also showed improved architecture, with the main changes occurring in the granular cell layer, which was similar to the control group (Figure 5B).

## 4 Discussion

Obesity is a significant global health issue that is linked to a rise in the prevalence of several diseases, including some types of cancer, diabetes, hypertension, cardiovascular diseases, and psychological or psychiatric disorders (Celik and Yildiz 2020; Monda et al. 2017). One of the safest and most effective strategies for losing weight is combining nutrition, exercise, and behavioral techniques (Celik and Yildiz 2020). In recent years, extensive research has considered natural products such as carotenoids as an attractive alternative to pharmaceuticals for treating obesity and reducing its complications (Al‐thepyani et al. 2022). Dietary supplements are not suggested alone for obesity management, but they should act synergistically with exercise to facilitate obesity prevention and treatment. Regular exercise is also important to maintain a healthy weight and can minimize the negative effects of obesity, even if there is no weight loss (Petridou et al. 2019). In this study, the effects of lutein and/or physical exercises on rat brains fed with a HFD were evaluated.

In this study, rats fed an HFD were successfully made obese after 12 weeks. There was a significant increase in the body weight of all HFD groups compared with the control. After five more weeks of treatment with Lu and/or PE, rats in all groups except the HFD group had slower rates of weight gain and reduced body weights. Among the treated groups, the HFD + Lu + PE group had the lowest body weight. This implies that lutein and/or exercise may be able to slow the development of obesity. Our data has shown the positive effect of lutein treatment on body weight and fat accumulation which is consistent with the studies (Alatawi et al. 2023; Han et al. 2015). Moreover, regular exercise and physical activity can boost the body's ability to burn off stored energy, which can help people manage their weight and lose obesity (Yang et al. 2016). Reduced food intake or increased energy expenditure may be the cause of the decrease in body weight, which shows a negative state of energy expenditure (Stenman et al. 2014). Although no statistically significant differences were observed in food or energy intake among the treated groups, a slight tendency toward reduced food consumption was noted in the HFD + Lu, HFD + PE, and HFD + Lu + PE groups compared to the HFD group. Similar observations have been reported in previous studies suggesting that high‐fat feeding and physical activity may influence feeding behavior and energy balance (da Rocha et al. 2016; Baek et al. 2020). Moreover, regular exercise has been shown to mitigate certain metabolic complications associated with obesity, even in the absence of significant weight reduction (Petridou et al. 2019).

HFD induces the accumulation of visceral adipose tissue which is an important mediator in the development of obesity and its related complications. Therefore, we examined the change in visceral adipose weight among the experimental groups. In comparison to the HFD group, rats treated with lutein and/or exercise had remarkably lower visceral adipose weight compared to the HFD group which is compatible with the study of Han et al. (2015). Additionally, exercise was proven to be the most effective non‐pharmacological option to reduce excessive visceral fat deposition and associated consequences in obese adults (Zhang et al. 2021).

Type 2 diabetes can be brought on by excessive body fat buildup. As a result, the incidence of type 2 diabetes has increased along with the prevalence of obesity (Klein et al. 2022). The groups that consumed HFD in the current study had considerably higher blood glucose levels than the control; this result is in agreement with the studies (Alkhudhayri et al. 2021; Haque and Ansari 2018). However, the supplementation of lutein and/or performing exercise led to a significant decrease in blood glucose levels in the HFD‐fed groups, which is consistent with the recent study of Gopal et al. (2023) who treated mice with lutein and found that glucose levels were lowered. Also, performing exercise has proved to improve insulin sensitivity and glucose levels (Chang et al. 2020).

The serum lipid profile is a reliable indicator of the onset of illnesses connected to metabolic problems brought on by obesity (Che et al. 2019). A HFD has been linked to dyslipidemia, which is characterized by increased total TG, TC, LDL‐C, and VLDL‐C levels and reduced HDL‐C (Mamun et al. 2019). Our findings show that lutein and/or exercise improve lipid profile and may prevent dyslipidemia in rats fed with HFD. The TG, TC, LDL‐C, and VLDL‐C levels were high while HDL‐C levels were low in rats fed an HFD compared to the control. We found that HDL‐C levels were greater in all groups (HFD + Lu, HFD + PE, and HFD + Lu + PE) compared with the HFD group, while TG, TC, LDL‐C, and VLDL‐C levels were lowered, which is consistent with the results of other research (Ahn and Kim 2021; Narayanamurthy et al. 2018; Tuzcu et al. 2017). Furthermore, previous studies showed that lutein has anti‐obesogenic properties that include the promotion of fatty acid oxidation and energy expenditure (Hajizadeh‐Sharafabad et al. 2021; Han et al. 2015). Also, exercise boosts lipolysis, lowers adipocyte fatty acid absorption, and modifies the fatty acid content of adipose tissue (Mika et al. 2019).

Most often, obese people have elevated levels of leptin, a hormone generated from adipose tissue that controls energy balance and body weight by binding to a particular receptor in the brain (Sáinz et al. 2015). In the current study, rats fed an HFD exhibited significantly higher leptin levels compared with the control, which aligns with the prior study of Santos et al. (Santos et al. 2019). On the other hand, leptin levels were significantly lowered in all treated groups with Lu and/or PE compared to the HFD group. High leptin levels in obese people might contribute to low‐grade chronic inflammation, which may cause degenerative illnesses and autoimmune reactivity (Baz et al. 2022). The induction of inflammatory mediator is inhibited by lutein, which downregulates redox‐sensitive inflammatory signaling pathways that might stop inflammatory disorders like obesity (Nakamura and Sugiura 2022). Thus, there could be a relation between lutein and leptin. Also, through the dysregulated expression of adipokines produced by adipose tissue, exercise not only helps to reduce the quantity of adipose tissue but may also reduce the inflammation brought on by obesity (Chilibeck et al. 2014).

Obesity caused by HFD is known for inducing oxidative damage via lipid peroxidation by increasing malondialdehyde (MDA), reducing endogenous antioxidants, and lowering the activity of antioxidant enzymes such as SOD and CAT (El Ayed et al. 2017). In our study, MDA levels were considerably elevated in the HFD group, while the levels of CAT and SOD were remarkably decreased compared to the control group. After administration of lutein and/or physical exercise, the MDA levels were significantly decreased. Additionally, the levels of CAT and SOD were significantly increased in all groups (HFD + Lu, HFD + PE, and HFD + Lu + PE) compared to the HFD group. According to the present data, lutein and exercise may both reduce oxidative stress by boosting CAT and SOD activities and lowering MDA, which is compatible with the previous studies (Sahin et al. 2020; Tuzcu et al. 2017). Fatani et al. (2016), proved the chemical structure of lutein that elucidates its antioxidant properties, which are believed to play a critical role in its biological function by showing two hydroxyl groups, one on each side of the molecule (Fatani et al. 2016). It's also important to note that exercise may have a positive effect in reducing oxidative stress. Exercise has been shown to increase the body's natural antioxidant production, which helps shield cells from oxidative damage (Neubauer and Yfanti 2015).

Increased inflammatory mediators that are crucial for preserving energy balance are brought on by obesity (Guillemot‐Legris and Muccioli 2017). In the current study, the inflammatory marker (IL‐6) levels were significantly higher in the HFD group than in the control group. Compared to the HFD group, the lutein and/or exercise performance groups showed a substantial reduction in IL‐6 levels. Additionally, the lutein groups (HFD + Lu and HFD + Lu + PE) showed lower IL‐6 levels, which agree with previous research (Tuzcu et al. 2017). In rats with severe traumatic brain injury, lutein reduced IL‐6 levels, protecting them from further damage (Tan et al. 2017). Additionally, regular exercise can lower the levels of IL‐6 in the elderly (Monteiro‐Junior et al. 2018).

Obesity lowers cognition and causes atrophy in the brain areas responsible for learning and memory (Bocarsly et al. 2015). In the present study, the Y maze was frequently utilized to evaluate learning and short‐term spatial working memory (Ullah et al. 2020). Moreover, the short‐term spatial memory task indicated spontaneous alternation (SA). A smaller percentage of SA activity is associated with memory impairment (Bahaidrah et al. 2022). Our result shows that SA was considerably decreased in the HFD group compared to the control group, which is compatible with the study (Kim et al. 2016). On the other hand, after administering lutein and/or performing exercise for 5 weeks, the groups (HFD + Lu, HFD + PE, and HFD + Lu + PE) showed a remarkably elevated SA compared to the HFD group. In agreement with Wang et al. (2018) study earlier research has shown that increased serum lutein concentrations in humans are favorably correlated with various cognitive functions (Cannavale et al. 2019). Additionally, low‐intensity exercise is as effective as high‐intensity exercise in promoting lipolysis and preventing the decline of cognitive function (Bae 2020).

Obesity brought on by HFD compromises spatial memory in the hippocampus and affects its structure (Heyward et al. 2016). The hippocampus and other memory‐related brain areas are damaged by obesity, and cerebral atrophy occurs more quickly (Mestre et al. 2017). The hippocampus is known to play a significant role in learning and memory and is particularly vulnerable to inflammatory damage due to the high density of inflammatory mediator receptors in this region of the brain (Green and Nolan 2014). In this study, histological examination of the brain of HFD‐induced obesity rats revealed the presence of variable changes in different regions of the hippocampus. The main changes were seen in the pyramidal cell layer of Cornu Ammonis CA1 and CA3, which showed a disorganized arrangement. Many pyramidal cells showed degenerative changes where they lost their shape and appeared shrank with dark cytoplasm and condensed nuclei. The molecular (ML) and polymorphic (PmL) layers of the DG showed increased neuroglial cells with pericellular haloes and dilated blood capillaries compared to the control group, in consistency with earlier studies (Jiang et al. 2020; Wu et al. 2018). Furthermore, after administration of lutein and/or exercise performance for 5 weeks, the examination of the hippocampus for all groups (HFD + Lu, HFD + PE, and HFD + Lu + PE) showed more improvement of the histopathological changes as compared with the HFD group, but the group (HFD + Lu + PE) revealed the highest improvement of histopathological changes. The histopathological data of the (HFD + Lu + PE) group showed more or less normal thickness and arrangement of the pyramidal layer in CA1 and CA3 where most of the pyramidal cells appeared preserved in shape and showed vesicular nuclei, while the DG showed improved architecture. The other research showed lutein has anti‐amnesic characteristics by regulating lipid peroxidation in neural cells, inhibiting acetylcholine esterase, and lowering oxidative stress in the hippocampus (Patel et al. 2021). Also, aerobic exercise has beneficial impacts on brain structure and function, including adult hippocampal neurogenesis and learning (Nokia et al. 2016).

The observed improvements in metabolic and biochemical markers in our data may reflect modulation of interconnected metabolic, oxidative, and inflammatory pathways. The increase in SOD and CAT activities together with the decrease in MDA levels indicates improved antioxidant status. Such findings are compatible with the involvement of the nuclear factor erythroid 2‐related factor 2 (Nrf2) in the signaling pathway that regulates the expression of antioxidant defense genes (Ahn and Kim 2021). It is therefore plausible that lutein and/or exercise enhanced antioxidant capacity via Nrf2‐related mechanisms. The reduction in IL‐6 levels further suggests attenuation of inflammatory signaling. Lutein exerts anti‐inflammatory effects by suppressing multiple inflammatory signaling mediators, such as nuclear factor kappa B (NF‐κB) and subsequently their pro‐inflammatory genes, such as *tumor necrosis factor α (TNF‐α), IL‐6. Cyclooxygenase‐2 (COX‐2), interleukin‐1β (IL‐1β)*, and *inducible nitric oxide* synthase (*iNOS*) (Kim et al. 2008; Prathyusha et al. 2025). Moreover, regular exercise has been reported to reduce circulating inflammatory markers in obese populations (Guo et al. 2024). Therefore, lowering oxidative stress and inflammation may have contributed to improved insulin sensitivity and glucose uptake by peripheral tissues, a well‐established consequence of reduced metabolic stress in obesity (Klein et al. 2022) that could explain the reduction in blood glucose level following lutein intervention and physical exercise. In addition, improvements in leptin levels may reflect enhanced leptin sensitivity, which is commonly impaired in diet‐induced obesity (Sáinz et al. 2015). Furthermore, the antioxidant and anti‐inflammatory properties of lutein may contribute to the observed decreases in LDL‐C, VLDL‐C, cholesterol and triglycerides through the attenuation of oxidative stress by suppressing nicotinamide adenine dinucleotide phosphate (NADPH) oxidase (NOX) activity that produces reactive oxygen species. (ROS); thereby, reducing lipid peroxidation and improving lipid metabolism (Eom et al. 2023; Han et al. 2015). It has also been reported that lutein supplement enhances β‐oxidation of fatty acids indirectly by up‐regulating gene expression of the nuclear receptor peroxisome proliferator‐activated receptor alpha (PPARα) in apolipoprotein E‐deficient mice feeding HFD; Therefore, it decreases adipocyte hypertrophy and attenuates adipose tissue inflammation (Han et al. 2015). The increased SOD and CAT activities may be explained by two possible mechanisms. First, lutein and/or exercise may enhance transcription of antioxidant enzyme genes through Nrf2 activation, thereby promoting antioxidant defense at the gene expression level (Ahn and Kim 2021). Second, reduced oxidative stress may preserve enzyme activity by limiting oxidative modifications that impair catalytic function, a process commonly associated with oxidative damage in obesity‐related conditions (Mullins et al. 2020). Improvements in behavioral performance and preservation of hippocampal structure indicate a protective effect on brain function. Exercise has been widely linked to enhanced neuroplasticity and brain health through mechanisms involving synaptic plasticity and neurotrophic signaling pathways, including brain‐derived neurotrophic factor (BDNF) (Cabral et al. 2019). Lutein, as a potent antioxidant, may further support hippocampal integrity by reducing oxidative stress and neuroinflammatory burden. (Tan et al. 2017), processes known to impair cognitive function in obesity (Mullins et al. 2020).

The combined reduction in oxidative stress and inflammatory mediators observed in the present study may therefore have contributed to the anti‐obesity effect, the preservation of hippocampal structure and the improvement of behavioral performance. Although lutein and physical exercise. Each independently improved the measured parameters, the combined intervention showed slightly greater improvements, suggesting complementary rather than strictly synergistic effects.

The present study provides important evidence for the protective effects of lutein and physical exercise, individually and in combination, against HFD‐induced metabolic, behavioral, and histopathological alterations. However, this study has several limitations that should be acknowledged. The limited sample size and intervention period may affect the generalizability of the findings. Moreover, the study focused on selected biochemical and behavioral parameters which may not fully capture the complexity of the underlying mechanisms. Future research should aim to investigate broader molecular mechanisms and incorporate additional behavioral assessments to better evaluate the long‐term effects of this combined approach on memory and cognitive function. In addition, comparative analyses of various carotenoids are warranted to determine those with superior neuroprotective efficacy, particularly when combined with physical exercise.

## 5 Conclusion

In conclusion, the present study demonstrates that combining lutein supplementation with physical exercise provides greater protection against HFD–induced metabolic and brain alterations than either intervention alone. This action is achieved by improving brain health, ameliorating adiposity, lipid profile, and glucose, and attenuating oxidative stress and inflammatory response induced by HFD in obese rats. Therefore, integrating nutritional and physical strategies may represent an effective approach to reduce obesity‐associated brain dysfunction.

## Author Contributions

**Hanna M. Alhoraibi:** conceptualization, validation, resources, data curation, supervision, project administration, writing – review and editing. **Saraa A. Almutairi:** methodology, software, investigation, resources, writing – original draft, visualization, funding acquisition, formal analysis.

## Funding

This project was funded by KAU Endowment (WAQF) at King Abdulaziz University, Jeddah, Saudi Arabia. The authors, therefore, acknowledge with thanks WAQF and the Deanship of Scientific Research (DSR) for technical and financial support.

## Ethics Statement

The animal study protocol was approved by the Ethics Committee of King Abdulaziz University (Approval No. ACUC‐21‐09‐37) and followed the rules and regulations of the Animal Care and Use Committee (ACUC) at the King Fahd Medical Re‐search Center (KFMRC).

## Conflicts of Interest

The authors declare no conflicts of interest.

## Supporting information


**Table S1:** Summary of metabolic and biochemical parameters in the experimental groups.

## Data Availability

The data that support the findings of this study are available from the corresponding author upon reasonable request.
