# Supplementary material for: Combined Lutein and Exercise Intervention Alleviates Brain Alteration Induced by a High‐Fat Diet in Obese Rats
Source: Food Sci Nutr. 2026 May 22;14(5):e71913. doi: 10.1002/fsn3.71913 (PMC13239562; doi:10.1002/fsn3.71913)
Supplement: Supplementary file 1 — Table S1: Summary of metabolic and biochemical parameters in the experimental groups. [file FSN3-14-e71913-s001.docx]

| **Parameters** | **Groups** | | | | |
| --- | --- | --- | --- | --- | --- |
|  | **Control** | **HFD** | **HFD + Lu** | **HFD + PE** | **HFD + Lu + PE** |
| **Leptin** | 38.17± 7.12 | 61.71±12.13  #### | 46.97±7.81  ** | 44.03±7.35 *** | 42.14 ± 4.77  **** |
| **TG** | 0.44 ± 0.09 | 0.97± 0.34  #### | 0.68 ± 0.11  # , ** | 0.77±0.13  ## | 0.59 ± 0.08  *** |
| **TC** | 1.02 ± 0.11 | 1.94 ± 0.18  #### | 1.55 ± 0.16  #### , ** | 1.62 ± 0.34  #### , * | 1.43 ± 0.21  ## , **** |
| **HDL-C** | 0.77 ± 0.12 | 0.37 ± 0.09  #### | 0.66 ± 0.12  **** | 0.64 ± 0.12  **** | 0.70 ± 0.11  **** |
| **LDL-C** | 0.13 ± 0.03 | 0.26 ± 0.04  #### | 0.13 ± 0.03  **** | 0.15 ± 0.04  **** | 0.11 ± 0.02  **** |
| **VLDL-C** | 0.09 ± 0.02 | 0.19 ± 0.07  #### | 0.14 ± 0.02  # , ** | 0.15 ± 0.03  ## | 0.12 ± 0.01  *** |
| **Blood Glucose** | 5.96 ± 0.52 | 11.92 ± 3.69  #### | 6.24 ± 0.73  **** | 6.88 ± 1.28 **** | 6.36 ± 1.15  **** |
| **MDA** | 0.20 ± 0.04 | 0.30 ± 0.03  #### | 0.23 ± 0.03  **** | 0.22 ± 0.02  **** | 0.22 ± 0.01  **** |
| **CAT** | 162.80 ± 32.20 | 147.20 ±23.10 | 307.70 ± 34.67  #### , **** | 219.80 ± 58.87 | 502.30 ± 106.80 #### , **** |
| **SOD** | 122.80 ± 9.01 | 64.77 ± 5.88  ### | 245.10 ± 35.52  #### , **** | 162.50 ±13.10  # , **** | 260.10 ± 48.63  #### , **** |
| **IL-6** | 60.19 ± 12.16 | 71.56 ± 9.70  # | 39.69 ± 5.61  #### ,**** | 62.50 ± 4.56 | 37.81 ± 6.31  #### , **** |

Data are presented as mean ± SD. Rats were divided into five groups (Control, HFD, HFD + PE, HFD + Lu, and HFD + Lu + PE) after for 17 weeks (n = 10 rats/group). Significant at ^#^ represents *P* < 0.05, ^##^ represents *P* <0.01, ^####^ *P* < 0.0001, ^####^ represents *P* <0.0001 when compared to the control; ^*^ represents *P* <0.05, ^**^ represents *P* <0.01, ^***^ represents *P* < 0.001, and ^****^ represents *P* <0.0001 when compared to the HFD groups.

 Supplementary Table S1. Summary of metabolic and biochemical parameters in the experimental groups.
